# The prognostic value of different glucose abnormalities in patients with acute myocardial infarction treated invasively

**DOI:** 10.1186/1475-2840-11-78

**Published:** 2012-06-28

**Authors:** Michal Mazurek, Jacek Kowalczyk, Radoslaw Lenarczyk, Teresa Zielinska, Agnieszka Sedkowska, Patrycja Pruszkowska-Skrzep, Andrzej Swiatkowski, Beata Sredniawa, Oskar Kowalski, Lech Polonski, Krzysztof Strojek, Zbigniew Kalarus

**Affiliations:** 1 Department of Cardiology, Congenital Heart Diseases and Electrotherapy, Medical University of Silesia, Silesian Center for Heart Diseases, ul. Szpitalna 2, 41–800, Zabrze, Poland; 2 Third Department of Cardiology, Medical University of Silesia, Silesian Center for Heart Diseases, Zabrze, Poland; 3 Department of Internal Diseases, Diabetology and Cardiometabolic Diseases, Medical University of Silesia, Silesian Center for Heart Diseases, Zabrze, Poland

**Keywords:** Acute myocardial infarction, Glucose abnormalities, Diabetes mellitus, Percutaneous coronary intervention

## Abstract

**Background:**

Diabetes (DM) deteriorates the prognosis in patients with coronary heart disease. However, the prognostic value of different glucose abnormalities (GA) other than DM in subjects with acute myocardial infarction (AMI) treated invasively remains unclear.

**Aims:**

To assess the incidence and impact of GA on clinical outcomes in AMI patients treated with percutaneous coronary intervention (PCI).

**Methods:**

A single-center, prospective registry encompassed 2733 consecutive AMI subjects treated with PCI. In all in-hospital survivors (n = 2527, 92.5%) without the history of DM diagnosed before or during index hospitalization standard oral glucose tolerance test (OGTT) was performed during stable condition before hospital discharge and interpreted according to WHO criteria. The mean follow-up period was 37.5 months.

**Results:**

The incidence of GA was as follows: impaired fasting glycaemia - IFG (n = 376, 15%); impaired glucose tolerance - IGT (n = 560, 22%); DM (n = 425, 17%); new onset DM (n = 384, 15%); and normal glucose tolerance – NGT (n = 782, 31%). During the long-term follow-up, death rate events for previously known DM, new onset DM and IGT were significantly more frequent than those for IFG and NGT (12.3; 9.6 and 9.4 vs. 5.6 and 6.4%, respectively, P < 0.05). The strongest and common independent predictors of death in GA patients were glomerular filtration rate < 60 ml/min/1,73 m^2 (HR 2.0 and 2.8) and left ventricle ejection fraction < 35% (HR 2.5 and 1.8, all P < 0.05) respectively.

**Conclusions:**

Glucose abnormalities are very common in AMI patients. DM, new onset DM and IGT increase remote mortality. Impaired glucose tolerance bears similar long-term prognosis as diabetes.

## Introduction

The prevalence of type 2 diabetes mellitus (DM) has rapidly increased worldwide over the last decades and DM is increasingly perceived as an ongoing epidemic. There is a conclusive evidence implicating DM in complications of coronary heart disease (CHD) [1], [2], [3] and [4]. The risk of acute myocardial infarction (AMI) and death in diabetic subjects without CHD is similar to the risk of non-diabetic pts with previous AMI. Therefore, DM has gained the status of CHD risk equivalent [5]. Despite recent treatment improvements of AMI, patients with DM have worse prognosis after myocardial infarction in comparison to subjects without DM. There is also evidence that CHD patients have glucose abnormalities (GA) other than DM. Mozaffarian et al. as well as Bartnik et al. compared population-based cohorts of patients with AMI and found that AMI groups showed almost twice higher annual incidence-rates of impaired fasting glucose and diabetes. Therefore, they proposed that myocardial infarction may be a prediabetes equivalent [6] and [7]. However, the early and long-term outcome in AMI patients with glucose abnormalities other than DM remains unclear. The aim of our study was to assess the incidence and prognostic role of different glucose abnormalities in AMI patients treated with percutaneous coronary intervention (PCI) as well as to identify independent predictors of death in GA.

## Materials and methods

### Data acquisition

A computerized database was used for prospective collection of data from 2733 consecutive patients admitted with AMI to our department. Recorded data included demographic and laboratory parameters, concomitant diseases, characteristics of AMI, types of glucose abnormalities, angiographic findings, outcomes of revascularization procedure, in-hospital complications and mortality. Data concerning long-term outcome was collected from a database of the National Fund of Health. The mean follow-up period was 37.5 months and the data was collected from 99% of patients enrolled in the study.

### Protocol of the registry

**Figure 1:**
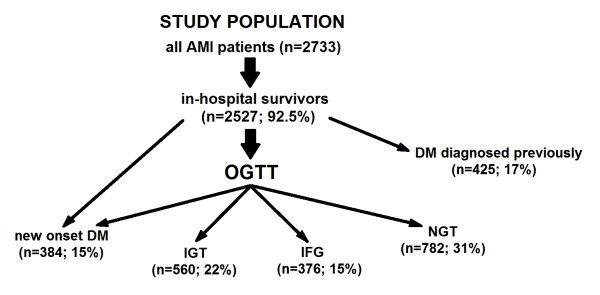
Study population selection, AMI – acute myocardial infarction, OGTT – oral glucose tolerance test, DM new onset – newly diagnosed diabetes mellitus, DM – diabetes mellitus diagnosed previously, IGT – impaired glucose tolerance, IFG – impaired fasting glucose, NGT – normal glucose tolerance.

The study population consisted of 2733 consecutive patients admitted to our Department with AMI and treated in the acute phase with PCI between January 2003 and December 2007. In all in-hospital survivors (n = 2527, 92.5%) without the history of diabetes mellitus diagnosed before or during index hospitalization standard oral glucose tolerance test (OGTT) was performed after stabilization of patients’ condition, routinely the day before or on discharge day, however no sooner than on day 5. Results of OGTT were interpreted in line with WHO recommendations for GA diagnosis [8]. This measure made it possible to diagnose the following glucose abnormalities: *IFG* - impaired fasting glycaemia; *IGT* - impaired glucose tolerance; *DM* - diabetes mellitus diagnosed previously; *new onset DM* - diabetes mellitus diagnosed during index hospitalization; and *NGT* - patients with normal glucose tolerance. Patients with IFG and IGT comprised a *prediabetic group*, while subjects with both, previously diagnosed DM as well as with the new onset DM represented the diabetic group. The rest of the patients, without any glucose disturbances, constituted the NGT group [Figure 1.

### Measurements and definitions

Diagnosis of glucose abnormalities was based on WHO criteria for venous plasma [8]. Patients were classified as having DM if they reported current or previous use of antidiabetic medications (insulin or oral hypoglycaemic agents). A new onset DM was diagnosed if fasting glycaemia during hospitalization, but no sooner than on day 5 was ≥7.0 mmol/l on at least two occasions (17.7% of cases) as well as on the basis of OGTT if fasting glycaemia was ≥7.0 mmol/l or a two hour post-load glucose level ≥11.1 mmol/l. OGTT made it possible to diagnose the new onset DM in 82.3% of cases. IFG was diagnosed if fasting glucose was ≥6.1 but <7.0 mmol/l, and IGT if fasting glucose was <7.0 mmol/l and postprandial glucose level ≥7.8 but <11.1 mmol/l. Normal glucose tolerance group consisted of patients with fasting glucose <6.1 mmol/l, and 2 h post-load glucose level <7.8 mmol/l.

Clinical AMI criteria evaluated on admission were: chest pain persisting > 20 min, ST segment elevation of at least 0.1 mV in two or more continuous ECG leads or non-diagnostic ECG with enzymatic confirmation of AMI. The biochemical criterion of myocardial infarction was elevated troponin I above the upper limit of normal. The other biochemical markers of myocardial injury: creatinine kinase (CK) and its isoenzyme MB (CK-MB) were assessed in all subjects. Patients were mainly admitted from referral hospitals and previous administration of fibrinolytic treatment was allowed. No upper age limit was used.

Duration of chest pain was estimated by the time interval between chest pain onset and the time of arrival to the emergency room. Multivessel disease was defined as the presence of >2 major epicardial coronary arteries or their major branches with stenosis of at least 70%, assessed during initial coronary angiography. Complete revascularization was defined when no total occlusion and no residual stenosis >70% (for left main >50%) was found in any major coronary artery or their major branches at discharge.

The estimated glomerular filtration rate was calculated using serum creatinine value on admission before catheterization, according to the abbreviated Modification of Diet in Renal Disease Study Group Equation proposed by National Kidney Foundation. Contrast-induced nephropathy (CIN) was defined as a rise in serum creatinine of 44.2 μmol/L (0.5 mg/dL), or a 25% increase from the baseline value within 48 h after PCI [9].

### Treatment protocol

In all consecutive patients, coronary angiography and PCI of infarct-related artery (IRA) were performed with the use of standard techniques immediately after hospital admission. All patients before coronary angiography received a single dose of oral aspirin (300–500 mg) and 5000–10000 U of intravenous heparin (additional boluses were given as appropriate to achieve activated clotting time >250 ms). The goal of PCI was to restore Thrombolysis in Myocardial Infarction (TIMI) 3 grade flow with residual stenosis lower than 30%, which denotes a successful procedure. After the intervention, all patients received 150 mg of aspirin daily indefinitely, 300 mg clopidogrel just before PCI, followed by 250 mg ticlopidine twice daily or 75 mg clopidogrel daily orally, as well as beta-blockers, ACE inhibitors/angiotensin receptor blockers (ARB) and statins, if these agents had not been contraindicated.

Hypoglycaemic treatment in the acute phase of AMI was in line with the DIGAMI protocol [10]. In patients with DM and blood glucose >11 mmol/l as well as in those without the previous diagnosis of DM, but with glucose levels over 11 mmol/l the insulin infusion was administered for at least 24 h followed by daily subcutaneous insulin injections for the remainder of the hospital stay and a minimum of 3 months thereafter. Every patient with the diagnosis of any type of GA was consulted by a diabetology specialist before hospital discharge and several issues with regard to lifestyle modification (body weight normalization, physical activity, smoking cessation), diet and medication were addressed.

### Outcomes

The primary outcome was death from any cause. Secondary outcomes included one of the following events: either recurrent myocardial infarction, repeated PCI, coronary artery by-pass grafting or stroke. Major adverse cardiovascular event (MACE) was defined as the occurrence of death or any of the above during observation period.

### Statistical analysis

Continuous parameters were expressed as means with standard deviations unless otherwise specified, categorical variables were presented as numbers and percentages. Comparative analysis between groups was performed using Student’s t-test for continuous variables and Chi-square or Fisher’s exact test, as appropriate, for dichotomous parameters. Log-rank tests were used to compare Kaplan-Meier curves plotted for cumulative survival and freedom from MACE. Independent predictors of death were identified with multivariate Cox-regression model and expressed as hazard ratio with 95% confidence interval. Regression models were developed after the inclusion of all parameters with significant univariate association with appropriate end-point. All tests were double-sided. P value <0.05 was considered statistically significant. All analyses were performed using the software package Statistica (version 6.1, StatSoft Inc., Tulsa, OK, USA).

The following variables were incorporated into the multivariate analysis model in order to identify independent predictors of death in different glucose abnormalities: age > 70 years, gender, prior stroke, prior AMI, previous CABG, previous PCI, multivessel disease, Killip class and cardiogenic shock on admission, hypertension, hyperlipidaemia, smoking, symptoms duration, IIb/IIIa inhibitors, unsuccessful PCI of IRA (TIMI < 3 after PCI), previous thrombolytic treatment, incomplete revascularization (ICR), glomerular filtration rate (GFR), contrast induced nephropathy (CIN), left ventricle ejection fraction (LVEF), type of AMI (NSTEMI vs STEMI), type of infarct-related artery (IRA), haematocrit, hemoglobin level.

### Ethics

All clinical data was obtained as the result of diagnostic and therapeutic procedures, which were in line with treatment guidelines for myocardial infarction. All patients provided an informed, written consent for hospitalization, invasive treatment and the use of collected data for research purposes.

## Results

### Baseline characteristics

Patients with DM and IGT were older, with lower frequency of male gender and smokers, more often presented with hypertension, chronic kidney disease and contrast-induced nephropathy. What is more, they had higher Killip class and higher values of admission glycaemia as well as lower ejection fraction and glomerular filtration rate in comparison to IFG and NGT groups.

Diabetic patients presented also with longer symptoms duration, higher frequency of prior AMI, multivessel coronary artery disease, incomplete revascularization, as well as lower efficacy of PCI defined as TIMI flow < 3 of IRA in comparison to other study groups.

The usage of beta-blockers, statins, ACE inhibitors/ARB, acetylsalicylic acid, and clopidogrel/ticlopidine during in-hospital period and prescribed at discharge did not differ significantly between study groups [Table 1].

**Table 1:** Comparative analysis of demographic, clinical and laboratory data among study groups

**Variable**	**DM (n = 425)**	**New onset DM (n = 384)**	**IGT (n = 560)**	**IFG (n = 376)**	**NGT (n = 782)**
Age (yrs)	65.5 ± 9.5*	65.3 ± 10.4*	62.3 ± 10.3*	58.2 ± 11.0	57.0 ± 11.3
Male – no. (%)	241 (56.7)*	237 (61.6)*	395 (70.5)*	316 (82.4)	618 (79.0)
Smoking – no. (%)	166 (39.1)*	182 (47.4)*	319 (56.9)*	245 (65.1)	533 (68.2)
Hypertension – no. (%)	320 (75.3)*	218 (56.9)*	302 (53.9)*	167 (44.3)	325 (41.6)
Hyperlipidaemia – no. (%)	232 (54.5)	200 (52.1)	295 (52.6)	208 (55.2)	411 (52.5)
Peripheral vascular disease – no. (%)	42 (9.9)	41 (10.8)	48 (8.5)	23 (6.1)	61 (7.8)
Creatinine on admission (μmol\L)	104.3 ± 86.6*	92.1 ± 39.2*	85.9 ± 34.0	80.3 ± 18.7*	85.5 ± 42.5
GFR (mL/min/1.73 m^2^)	73.3 ± 26.7*	77.6 ± 25.6*	84.5 ± 25.2*	92.5 ± 24.5*	89.2 ± 25.6
Contrast-induced nephropathy – no. (%)	110 (25.8)*	127 (33.2)*	150 (26.8)*	79 (21.1)	133 (17.0)
Previous myocardial infarction – no. (%)	113 (26.5)*	75 (19.6)	95 (16.9)	61 (16.3)	132 (16.9)
Previous CABG – no. (%)	16 (3.8)	10 (2.7)	12 (2.2)	10 (2.7)	26 (3.3)
Previous PCI – no. (%)	63 (14.8)*	31 (8.2)	54 (9.6)	26 (7.0)	66 (8.4)
Glucose on admission (mmol/L)	12.2 ± 5.4*	9.6 ± 4.1*	7.8 ± 2.5*	7.2 ± 2.0	7.2 ± 2.2
Pain duration (hours)	7.9 ± 9.9*	7.9 ± 10.5*	5.8 ± 6.6	5.7 ± 5.9	5.9 ± 7.4
Killip-class on admission	1.3 ± 0.7*	1.4 ± 0.8*	1.3 ± 0.6*	1.2 ± 0.5	1.2 ± 0.5
Anterior infarction – no. (%)	144 (33.8)	150 (39.1)*	195 (34.8)	143 (37.9)*	242 (31.0)
Ejection fraction (%)	42.0 ± 8.4*	42.8 ± 8.3*	43.8 ± 7.8*	44.9 ± 7.7	45.7 ± 7.0
Fibrinolysis – no. (%)	10 (2.3)	10 (2.5)	21 (3.8)	8 (2.1)	31 (4.0)
GP IIb/IIIa – inhibitor – no. (%)	68 (16.0)*	66 (17.3)*	80 (14.3)	58 (15.5)*	84 (10.8)
Beta-adrenergic blocker – no. (%)	372 (87.6)	338 (88.1)	498 (88.9)	327 (87.0)	702 (89.8)
ACE-inhibitor/ARB – no. (%)	369 (86.9)	328 (85.4)	483 (86.2)	320 (85.2)	672 (85.9)
Aspirin – no. (%)	414 (97.4)	376 (98.0)	549 (98.0)	369 (98.2)	769 (98.3)
Statin – no. (%)	357 (83.9)	323 (84.0)	471 (84.1)	312 (83.1)	658 (84.2)
Clopidogrel/Ticlopidine – no. (%)	369 (86.9)	335 (87.2)	488 (87.1)	325 (86.4)	683 (87.4)
Multivessel coronary artery disease –no (%)	322 (75.8)*	252 (65.7)*	346 (61.7)	222 (59.1)	456 (58.3)
Incomplete revascularization – no. (%)	240 (56.5)*	177 (46.1)	264 (47.1)	172 (45.8)	328 (41.9)
TIMI flow <3 after PCI of IRA – no. (%)	63 (14.9)*	70 (18.1)*	72 (12.8)	45 (12.1)	77 (9.8)
Hospitalization time (days)	9.8 ± 7.9*	10.3 ± 6.2*	8.9 ± 4.6*	7.7 ± 2.6	7.6 ± 4.3

Values presented as means ± SD or percentage of subjects. ARB = angiotensin receptor blockers; CABG = coronary artery by-pass grafting; DM = type 2 diabetes mellitus; GFR = glomerular filtration rate; GP IIb/IIIa = glycoprotein IIb/IIIa; IGT = impaired glucose tolerance; IFG = impaired fasting glucose; NGT = normal glucose tolerance; IRA = infarct-related artery; PCI = percutaneous coronary intervention; TIMI = Thrombolysis in Myocardial Infarction.

* – p value <0.05 compared with normal glucose tolerance group (NGT).

### Long term outcome

Total mortality rate in the entire study population was 8.6%. There were no differences in 30-day mortality between all study groups. However, during 1-year follow-up death rate events were significantly more frequent both in DM and IGT in comparison to IFG and NGT groups (9.0 and 6.7 vs. 2.9 and 3.7%, respectively, P < 0.05). Similarly, during the long-term follow-up, death rate events for previously known DM, new onset DM and IGT were also significantly more frequent than those for IFG and NGT groups (12.3; 9.6 and 9.4 vs. 5.6 and 6.4%, respectively, P < 0.05) [Table 2].

**Table 2:** Comparative analysis of the outcomes between study groups

	**DM (n = 425)**	**New onset DM (n = 384)**^**a**^	**IGT (n = 560)**^**b**^	**IFG (n = 376)**^**c**^	**NGT (n = 782)**^**d**^
30-day outcome:					
Myocardial infarction – no. (%)	4 (1.0)	5 (1.3)	10 (1.8)	6 (1.7)	13 (1.7)
PCI – no. (%)	16 (3.7) ^b^	6 (1.6)	7 (1.3)	9 (2.4)	18 (2.3)
CABG – no. (%)	0 (0)	4 (1.0) ^d^	4 (0.7) ^d^	1 (0.3)	0 (0.0)
Stroke – no. (%)	1 (0.2)	1 (0.3)	1 (0.2)	2 (0.5)	2 (0.3)
Mortality – no. (%)	9 (2.1)	3 (0.8)	6 (1.0)	2 (0.5)	9 (1.1)
MACE – no. (%)	28 (6.6) ^c d^	15 (4.0)	23 (4.1)	12 (3.2)	26 (3.3)
1-year outcome:					
Myocardial infarction – no. (%)	63 (14.8)	42 (11.0)	86 (15.3)	59 (15.8)	106 (13.6)
PCI – no. (%)	90 (21.2) ^b^	72 (18.8)	86 (15.3)	70 (18.6)	129 (16.5)
CABG – no. (%)	41 (9.6)	32 (8.3)	54 (9.7)	41 (10.9)	64 (8.2)
Stroke – no. (%)	7 (1.6)	9 (2.3) ^d^	8 (1.4)	5 (1.3)	5 (0.6)
Mortality – no. (%)	38 (9.0) ^a c d^	20 (5.2)	38 (6.7) ^c d^	11 (2.9) ^b^	29 (3.7)
MACE – no. (%)	174 (41.0) ^a c d^	111 (29.0) ^b^	208 (37.1) ^a c d^	113 (30.0) ^b^	229 (29.3)
Remote follow up:					
Myocardial infarction – no. (%)	73 (17.2)	51 (13.3)	95 (17.0)	63 (16.8)	128 (16.4)
PCI – no. (%)	108 (25.4) ^b^	88 (22.9)	103 (18.4)	79 (21.0)	166 (21.2)
CABG – no. (%)	49 (11.5)	32 (8.3)	55 (9.9)	46 (12.2)	72 (9.2)
Stroke – no. (%)	13 (3.1)	12 (3.1)	11 (2.0)	8 (2.1)	11 (1.4)
Mortality – no. (%)	52 (12.3) ^c d^	37 (9.6) ^c d^	53 (9.4) ^c d^	21 (5.6) ^a b^	50 (6.4)
MACE – no. (%)	187 (43.9)	153 (39.8)	227 (40.6)	152 (40.4)	310 (39.7)

Values presented as percentage of subjects. CABG = coronary artery by-pass grafting; DM = type 2 diabetes mellitus; IGT = impaired glucose tolerance; IFG = impaired fasting glucose; NGT = normal glucose tolerance; MACE = major adverse cardiovascular event; PCI = percutaneous coronary intervention.

^a^ – p value <0.05 compared with new onset DM group.

^b^ – p value <0.05 compared with IGT group.

^c^ – p value <0.05 compared with IFG group.

^d^ – p value <0.05 compared with NGT group.

**Figure 2:**
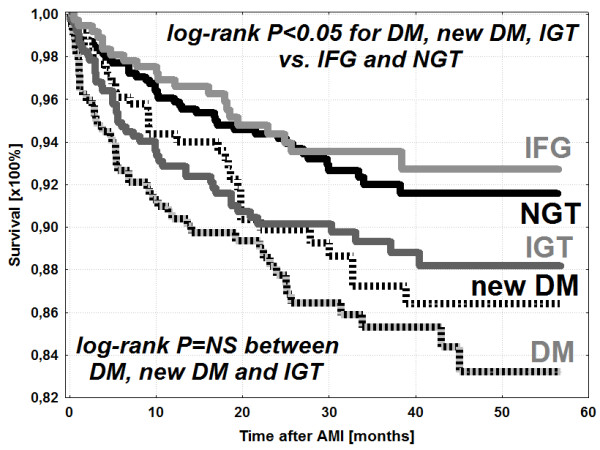
**Kaplan-Meier survival curves in particular study groups**,** NGT – normal glucose tolerance, New DM – newly diagnosed diabetes mellitus, DM – diabetes mellitus diagnosed previously, IGT – impaired glucose tolerance, IFG – impaired fasting glucose, IFG and control group vs IGT, new onset DM and DM; p < 0.05.** IFG vs control group; p = NS. IGT vs new onset DM vs DM; p = NS. Time in months.

Incidence of MACE was significantly higher in DM compared to other study groups in a 30-day follow-up. However, while in a 1-year observation period MACE events were more frequent in DM and IGT groups, during the remote observation there were no significant differences in MACE among all study groups [Table 2]. Cumulative survival rates in study groups with different GA have been presented in Figure 2.

### Independent predictors for death

The multivariate Cox-regression analysis revealed the independent predictors of death in GA patients [Table 3]. The strongest and common independent risk factors for death in both prediabetic and diabetic group were glomerular filtration rate < 60 ml/min/1,73 m^2 (HR 2.0 and 2.8) and left ventricle ejection fraction < 35% (HR 2.5 and 1.8, all P < 0.05) respectively.

**Table 3:** Independent risk factors for death

**Independent predictors of death**	**Prediabetic group**			**Diabetic group**		
	**HR**	**95% CI**	**p**	**HR**	**95%CI**	**p**
GFR < 60 ml/min/1,73 m^2	2.03	1.43-2.63	0.02	2.84	2.37-3.31	<0.01
LVEF < 35%	2.52	1.96-3.08	<0.01	1.82	1.29-2.35	0.029
Age > 70 yrs	2.20	1.7-2.7	<0.01	1.39	0.92-1.86	0.17

GFR - glomerular filtration rate.

LEVF – left ventricle ejection fraction.

## Discussion

Results of several cohort studies revealed that glucose abnormalities other than DM are very frequent in patients with CHD [11], [12] and [13]. The Euro Heart Survey on Diabetes and the Heart showed that the rates of normal glucose tolerance were less common than abnormal ones in patients with diagnosed CHD and that over 50% of CHD subjects had IGT or newly detected DM [12] and [14]. Our results confirmed the high prevalence of abnormal glucose metabolism in AMI patients, with only 31% of subjects with normal glucose tolerance and a high proportion of DM, IGT and IFG (32, 22 and 15% respectively).

Published data indicates also that hyperglycaemia is associated with adverse outcomes regardless of diabetes status [15], [16] and [17]. Stress hyperglycaemia in a setting of AMI increases the risk of malignant ventricular tachyarrhythmias as well as in-hospital mortality [16]. In the observational registry of consecutive nondiabetic patients with STEMI Timmer *et al.* reported that acute admission hyperglycaemia was associated with increased 1-year and remote mortality [17]. However, by means of elevated admission glucose as well as HbA_1c_ the authors were able to identify the first parameter as the predictor of early adverse outcomes, while the latter one was independently associated with 1-year and long-term mortality. Similarly, Chan et al. showed that HbA_1c_ concentration in diabetic patients in a setting of acute coronary syndrome is not related to short-term cardiovascular outcome [18].

It is known that patients with both CHD and DM have worse outcomes than subjects with only one of these two conditions [5], [19] and [20]. However, to the best of our knowledge, there is very limited data on the impact of different glucose perturbations on early and late clinical outcomes in AMI patients treated with PCI in the acute phase of myocardial infarction. Lenzen et al. in a population of coronary artery disease showed that patients with previously recognized DM are at highest risk for adverse events, while those with newly detected DM are at intermediate risk. Nevertheless, IFG and IGT were not identified as independent predictors of worse outcome. What is more, acute coronary syndromes accounted for only 36% of patients in this study [14]. In GAMI study, the analysis of AMI population with coexisting abnormal glucose tolerance revealed that both IGT and newly diagnosed DM are strong and independent predictors of adverse cardiovascular events after myocardial infarction [13]. However, of note is that the GAMI population was relatively small and consisted of only 168 subjects. The group with abnormal glucose tolerance included 113 patients not only with IGT, but also with a newly detected DM. What is more, reperfusion therapy had been used only in 38% of patients with glucose abnormalities and in 51% with normal glucose regulation.

Our registry encompassed 2527 consecutive in-hospital AMI survivors, who were optimally treated, both pharmacologically and invasively (reperfusion by means of PCI only) in the acute phase of AMI. Total mortality rate of 8.6% in our study was relatively low. However, this may be a result of the study protocol, which was based on the analysis of in-hospital survivors (n = 2527, 92.5%) in order to assess properly the outcomes of AMI patients with different glucose abnormalities, in particular with GA other than previously diagnosed DM. Indeed, it would be impossible to diagnose accurately and reliably GA other than previously diagnosed DM in patients who died during hospitalization. Among 206 subjects with AMI (n = 7.5%) who died during index hospitalization 107 (52%) subjects had previously diagnosed DM. What is more, all consecutive patients with AMI were treated very homogenously and optimally both pharmacologically and invasively with modern therapy (PCI), in line with ESC recommendations and this may have contributed to the relatively low long-term mortality.

The main finding of our study is that IGT affects negatively the outcomes of AMI patients treated invasively in the acute phase of myocardial infarction. What is more, the long-term prognosis of patients with IGT is similar to the outcomes of AMI subjects with DM and significantly worse than in normoglycaemic patients. Both, IGT and DM increase significantly one year and remote mortality in AMI population.

Moreover, further multivariate analysis model identified independent predictors of death in subjects with glucose abnormalities. Impaired renal function along with depressed left ventricle ejection fraction appeared to be the strongest and common risk factors for death in both prediabetic and diabetic groups. On a similar study population Kowalczyk et al. found that the prognosis of diabetics with AMI is related to renal function and diabetes coexisting with chronic kidney disease (CKD) is one of the strongest independent risk factors for cardiovascular complications and total mortality [21]. Similarly, Kim et al. in a nationwide prospective Korea Acute Myocardial Infarction Registry (KAMIR) showed that compromised renal function, in particular in combination with diabetes, is associated with the occurrence of composite MACE and indicates poor prognosis in subjects with AMI [22]. The impact of abnormal glucose metabolism on left ventricular function and prognosis in AMI patients was assessed by Høfsten et al. who demonstrated a linear association between GA and left ventricular dysfunction [23]. What is more, Juana A Flores-Le Roux et al. reported that in subjects with acute decompensation of heart failure, a new onset diabetes is not only common, but also increases remote mortality in the same manner as previously diagnosed diabetes [24].

Our results thus confirmed an observation, that large proportion of AMI patients has IGT as well as that this type of abnormal glucose metabolism indicates the group of patients at a high risk of adverse cardiac events. Therefore, interventions which would improve the prognosis in this group of AMI patients should be considered. Published data indicates that smoking cessation, prevention of weight gain, and consumption of low-fat dairy products could substantially lower this risk [6] and [25]. However, although AMI individuals are usually scheduled to follow these recommendations, our results suggest that this type of strategy is insufficient.

Although it has been suggested, that IGT may be a more “benign” state than DM the results of our registry indicate, that both IGT and DM bear poor prognosis in AMI patients [6]. The intriguing convergence of survival Kaplan-Meier curves in diabetic and IGT-patients who survived AMI may be explained by different treatment strategies in both groups. Diabetic patients, in contrast to IGT subjects, receive glucose-lowering medications during acute AMI phase and throughout the follow-up period. Consequently, the beneficial effect of such a pharmacotherapy only in DM subjects could be responsible for similar long-term prognosis in both groups.

Up to date, only a few studies examined the effects of antidiabetic treatment in IGT subjects [25], [26] and [27]. However, the STOPNIDDM trial revealed very optimistic results [25] and [26]. In this randomized, placebo-controlled trial Acarbose – oral anti-diabetic agent, significantly prevented or delayed the progression of glucose intolerance to diabetes and, most importantly, significantly reduced the risk of cardiovascular events. These data provided a strong rationale for the use of similar agents as a part of treatment strategy in AMI patients with coexisting IGT to improve long-term prognosis.

Some recently published trials also indicated that lifestyle modification and various pharmacological agents can delay or prevent the development of glucose disturbances to overt diabetes. However, the expected rates of cardiovascular events in these studies were low and did not provide statistical power to evaluate the influence of such interventions on MACE [28], [29] and [30]. The recently published, promising study in this area was The Navigator trial. The study was designed to evaluate whether reducing postprandial hyperglycaemia and blockade of the renin-angiotensin-aldosterone system or both interventions reduce the risk of diabetes and cardiovascular events in patients with IGT [31]. However, after a median follow up of 6.5 years, neither valsartan nor nateglinide improved cardiovascular prognosis in the study population. What is more, nateglinide did not reduce the risk of new onset diabetes, while valsartan reduced this risk only by 14% [32] and [33]. The above mentioned conflicting data from different studies indicates the need for further randomized clinical trials in order to establish the position of glucose-lowering agents in prevention of adverse cardiovascular events in AMI patients.

### Clinical implications

Our data confirmed the importance of OGTT performed before hospital discharge in detection of glucose abnormalities in non-diabetic AMI patients. Similarly to diabetic patients, subjects with IGT who survived myocardial infarction treated with PCI in the acute phase have unfavorable long-term prognosis. Further studies are necessary to evaluate the role of new, additional methods of treatment, possibly including glucose lowering agents to improve prognosis in this high-risk group of patients.

### Limitation of the study

This is a single center, observational registry. Differences in baseline characteristics among study groups are a result of the registry protocol, which allowed to enroll consecutive in-hospital AMI survivors who were treated with PCI in the acute phase of myocardial infarction. Neither OGTT was repeated in the follow-up period nor details regarding further hypoglycaemic treatment are available.

## Competing interests

The authors state, that they have read and approved the manuscript, the paper is original, has not been published and is not under simultaneous consideration for publication elsewhere, and none of the authors has any competing interest to disclose.

## Authors’ contributions

The corresponding author MM collection of data, and JK conception and design, analysis and interpretation of data, drafting of the manuscript, final approval of the manuscript submitted. RL, LP, KS, and ZK interpretation of data, revising manuscript critically for important intellectual content, final approval of the manuscript submitted. TZ, AS, PSP, SA, BS, OK collection of data, analysis and interpretation of data, final approval of the manuscript submitted. All authors read and approved the final manuscript.
